# Seco-sativene and Seco-longifolene Sesquiterpenoids from Cultures of Endophytic Fungus *Bipolaris eleusines*

**DOI:** 10.1007/s13659-016-0116-4

**Published:** 2017-01-06

**Authors:** Man-Si Yang, Xiao-Yue Cai, Yuan-Yuan He, Meng-Ying Lu, Shuang Liu, Wen-Xiang Wang, Zheng-Hui Li, Hong-Lian Ai, Tao Feng

**Affiliations:** 1 School of Agriculture and Biological Technic, Yunnan Agricultural University, Kunming, 650201 China; 2 College of Pharmacy, South-Central University for Nationalities, Wuhan, 430074 China

**Keywords:** *Bipolaris eleusines*, Sesquiterpenoid, Bipolenins D–F

## Abstract

Two new seco-sativene sesquiterpenoids, bipolenins D (**1**) and E (**2**), a new seco-longifolene sesquiterpenoid, bipolenin F (**3**), together with three known analogues (**4**–**6**), were obtained from cultures of endophytic fungus *Bipolaris eleusines*. Their structures were established by MS and NMR data. Compounds **1**–**6** showed no activity to five human cancer cell lines.

**Electronic supplementary material:**

The online version of this article (doi:10.1007/s13659-016-0116-4) contains supplementary material, which is available to authorized users.

## Introduction

**Fig. 1:**
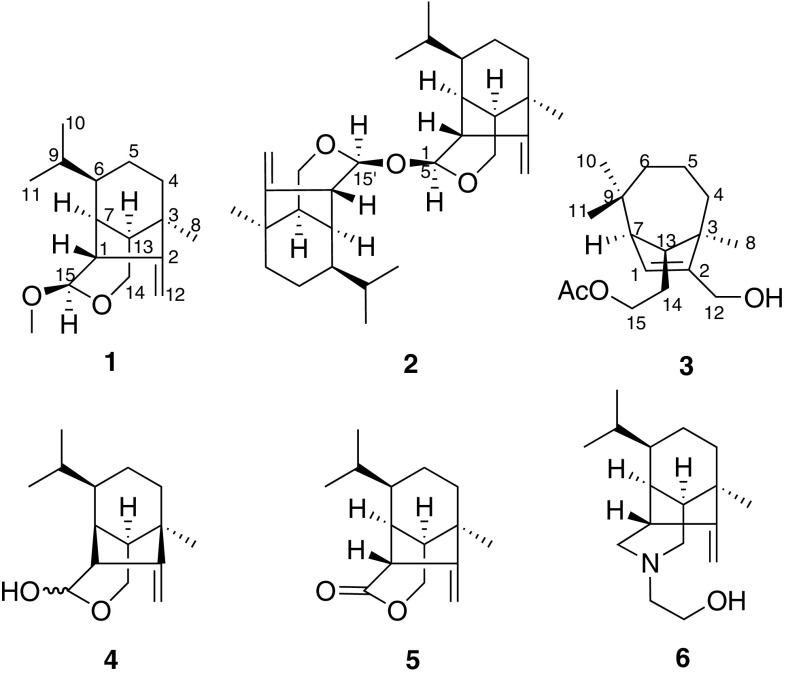
Structures of compounds **1**–**6**

Sativene sesquiterpenoids, as well as its derivatives, usually possessed an unusual backbone characterized by a 5/6 or 5/7 bridge ring system [1–7], which have become targets of synthetic chemists [8–12]. Previously, these sesquiterpenoids were obtained mainly from fungi *Cochliobolus*, *Drechslera*, and *Bipolaris*, et al., while some of them displayed bioactive diversity such as antiplasmodial activity and cytotoxicity [1–7, 13, 14]. We also reported sativene sesquiterpenoids bipolenins A–C from cultures of endophytic fungus *Bipolaris eleusines* [15], while bipolenin B showed certain cytotoxicities to human cancer cell lines [15]. In order to search for more interesting sativene sesquiterpenoids, we expanded the fermentation of *B. eleusines* and finished the chemical studies. As a result, we obtained three new sativene sesquiterpenoids, named bipolenins D–F (**1**–**3**), as well as three known ones, prehelminthosporol (**4**) [13], prehelminthosporolactone (**5**) [13, 14], and victoxinine (**6**) [14] (Fig. 1). Their structures were elucidated by extensive spectroscopic methods. Compound **2** was a dimer which was rarely found previously.

## Results and Discussion

Compound **1** was isolated as a colorless oil. The molecular formula was established as C_16_H_26_O_2_ by HREIMS at *m/z* 273.1818 [M + Na]^+^ (calcd for C_16_H_26_O_2_Na, 273.1825), corresponding to four degrees of unsaturation. The IR absorption bands at 1631, 1454, and 1368 cm^−1^ revealed the existence of double bond. In the ^1^H NMR spectrum of compound **1** (Table 1), signals at *δ*_H_ 0.85 (3H, d, *J* = 6.6 Hz, Me-10), 0.94 (3H, d, *J* = 6.6 Hz, Me-11), and 1.19 (3H, s, Me-8) were ascribable for three methyl groups, while signals at *δ*_H_ 4.82 and 4.72 should be assigned to a terminal double bond. The ^13^C NMR and DEPT spectra showed 16 carbon resonances which were ascribable for four methyl (including one methoxy), four methylene, six methine and two quaternary carbons (Table 1). On the basis of data comparison with those sesquiterpenoids reported from the same resource [13–15], compound **1** was suggested to possess a seco-sativene backbone with similar structure to that of prehelminthosporol (**4**) [13]. The key difference was that the hydroxy at C-15 in **4** was replaced by a methoxy group in **1**, as indicated by HMBC correlation from *δ*_H_ 3.36 (3H, s, OMe) to *δ*_C_ 103.8 (d, C-15). Detailed analyses of ^1^H–^1^H COSY and HMBC data suggested that other parts of **1** were the same to those of **4**. In addition, the ROESY correlation between H-15 and H-7 were detected, which indicated that H-15 should be *α* oriented. Therefore, the structure of compound **1** was established and named bipolenin D (Fig. 1).

**Table 1:** ^1^H and ^13^C NMR data of compounds **1**–**3** (CDCl_3_, *δ* in ppm, *J* in Hz)

No.	**1**		**2**		**3**	
	*δ*_H_	*δ*_C_	*δ*_H_	*δ*_C_	*δ*_H_	*δ*_C_
1	2.57, br s	48.6, d	2.60, br s	49.0, d	5.71, br s	127.4, d
2		158.1, s		157.8, s		146.5, s
3		43.9, s		43.8, s		50.3, s
4	1.45, m; 1.38 m	42.0, t	1.46, m; 1.39, m	41.9, t	1.62, m; 1.41, m	44.4, t
5	1.64, m; 1.11, m	25.9, t	1.66, m; 1.14, m	26.0, t	1.46, m; 1.36, m	21.0, t
6	1.08, m	46.7, d	1.12, m	46.7, d	1.34, m	41.3, t
7	2.61, br s	37.8, d	2.68, br s	37.9, d	1.94, dd (11.8, 4.0)	45.6, d
8	1.19, s	20.3, q	1.19, s	20.4, q	1.02, s	21.2, q
9	1.32, m	30.8, d	1.34, m	31.0, d		35.7, s
10	0.85, d (6.6)	21.0, q	0.87, s	21.2, q	0.89, s	31.9, q
11	0.94, d (6.6)	21.1, q	0.98, s	21.1, q	0.91, s	27.3, q
12	4.82, s; 4.72, s	101.1, t	4.85, s; 4.71, s	101.4, t	4.11, d (14.0)	59.8, t
					4.03, d (14.0)	
13	1.32, m	51.5, d	1.34, m	51.4, d	1.98, br s	57.0, d
14	3.74, br s	64.8, t	3.77, br s	65.1, t	1.76, m; 1.37, m	29.8, t
15	4.39, d (3.3)	103.8, d	4.79, d (3.0)	99.6, d	4.13, m; 3.99, m	63.7. t
O*Me*	3.36, s	54.6, q				
O*Ac*					2.05, s	21.0, q
						171.2, s

Compound **2** was isolated as a colorless oil. The molecular formula C_30_H_46_O_3_ was identified by HRESIMS at *m/z* 477.3340 [M + Na]^+^ (calcd for C_30_H_46_O_3_Na, 477.3345). The ^1^H NMR spectrum of **2** seemed to be similar to that of **1**, including three singlets for three methyl, as well as signals for a terminal double bond. The ^13^C NMR spectrum displayed fifteen carbon resonances comprised of three methyl, four methylene, six methine, and two quaternary carbons. These data suggested that compound **2** was a dimer of seco-sativene sesquiterpenoid, which possessed a completely symmetrical structure. Preliminary analyses of NMR data suggested that the two units of compound **2** were closely related to that of **1**. In the HMBC spectrum, a key correlation from *δ*_H_ 4.79 (1H, d, *J* = 3.0 Hz, H-15 and H-15′) to *δ*_C_ 99.6 (d, C-15′ and C-15) suggested that the two units were connected by ester bond of C-15–*O*–C-15′, which allowed the two units were completely the same. The ROESY correlations of H-15/H-7, H-15/H-14, and H-14/H-7, as well as similar coupling constant of H-15 (d, *J* = 3.0 Hz) with that in **1**, indicated that H-15 and H-15′ should be *α* oriented. Therefore, the structure of compound **2** was established and named bipolenin E.

Compound **3** was isolated as a colorless oil. The molecular formula was established as C_17_H_28_O_3_ by HREIMS at *m/z* 280.2025 [M]^+^ (calcd for C_17_H_28_O_3_, 280.2038). The ^13^C NMR spectrum displayed seventeen carbon resonances. Of them, signals at *δ*_C_ 21.0 (q) and 171.2 (s) were ascribed to one acetoxyl group, while other fifteen signals were similar to those of secolongifolene diol [5]. These data suggested that compound **3** should be an *O*-acetyl derivative of secolongifolene diol. The significant downfield shifts of CH-15 (*δ*_H_ 4.13 and 3.99; *δ*_C_ 63.7) indicated that the *O*-acetyl group should be placed at C-15. The other data suggested that other parts of **3** were the same to those of secolongifolene diol. Previously, we obtained *O*-acetyl derivatives of secolongifolene diol from the same resource, bipolenins B and C [15], in which the NMR data were also similar to those of **3** [15]. Therefore, compound **3** was established and named bipolenin F.

Three known sativene sesquiterpenoids, prehelminthosporol (**4**) [13], prehelminthosporolactone (**5**) [13, 14], and victoxinine (**6**) [14] were also obtained. Their structures were identified by comparison data with those reported in the literature.

Compounds **1**–**6** were evaluated for their cytotoxicities against five human cancer cell lines using the method reported previously [15]. However, no compound showed any activity (IC_50_ > 40 μM).

## Experimental Section

### General Experimental Procedures

Optical rotations were obtained on a JASCO P-1020 digital polarimeter (Horiba, Kyoto, Japan). NMR spectra were obtained on Bruker 500 MHz and Bruker Avance III 600 MHz spectrometers (Bruker, Karlsruher, Germany). HRESIMS were recorded on an Agilent 6200 Q-TOF MS system (Agilent Technologies, Santa Clara, CA, USA). HREIMS were measured on a Waters Autospec Premier P776 mass spectrometer (Waters, Milford, MA, USA). Silica gel 200–300 mesh (Qingdao Marine Chemical Inc., Qingdao, China) and Sephadex LH-20 (Amersham Biosciences, Upssala, Sweden) were used for column chromatography. Preparative High Performance Liquid Chromatography (Pre-HPLC) was performed on an Agilent 1260 liquid chromatography system equipped with a Zorbax SB-C18 column (5 μm, 9.4 mm × 150 mm) (Agilent Technologies, Santa Clara, CA, USA).

### Fungus Material

The fungus *Bipolaris eleusines* was isolated from fresh potatoes sampled from the nursery of Yunnan Agricultural University at random in July 2012. The fungus was identified by observing the morphological characteristics and analysis of the internal transcribed spacer (ITS) regions (Max identity: 99%; Query cover: 98%; Accession: KY909768.1). The strain is preserved at Yunnan Agricultural University, China (No. PE20120728-2).

### Fermentation, Extraction, and Isolation

This fungal strain was cultured on potato dextrose agar (PDA) medium at 25 °C for 10 days. The agar plugs were inoculated in 500-mL Erlenmeyer flasks, each containing 100 mL of potato dextrose media. Flask cultures were incubated at 28 °C on a rotary shaker at 160 rpm for two days as seed culture. Sixty 500-mL Erlenmeyer flasks each containing 150 mL of potato dextrose broth (PDB) were individually inoculated with 25 mL of seed culture, and were incubated at 25 °C on a rotary shaker at 160 rpm for 15 days.

The cultures of *B. eleusines* (40 L) were extracted four times by EtOAc to afford a crude extract (21 g), which was subjected to silica gel column chromatography, eluted with a gradient of CHCl_3_-MeOH (1:0 to 0:1) to obtain six fractions (A–F). Fraction B (4.4 g) was separated repeatedly by silica gel (petroleum ether (PE)-acetone; 10:1 to 5:1) to afford **1** (6.8 mg), **2** (6.4 mg), and **5** (3.8 mg). Fraction C (4.3 g) was eluted with PE-acetone (7:1 to 1:1) to give six sub-fractions (C1–C6). Fraction C2 (120 mg) was purified by pre-HPLC (MeCN/H_2_O from 40/60 to 80/20 in 20 min, flow speed: 10 mL/min) to afford **3** (1.3 mg), **4** (7.2 mg), and **6** (2.1 mg).

### Bipolenin D (**1**)

Colorless oil. [α]_D_^20^ −21.7 (*c* 0.20, CHCl_3_). IR (KBr) ν_max_ cm^−1^: 3428, 2920, 1631, 1454, 1368, 1122, 1063, 981, 881; ^1^H NMR (500 MHz, CDCl_3_) and ^13^C NMR (125 MHz, CDCl_3_) data, see Table 1.

### Bipolenin E (**2**)

Colorless oil. [α]_D_^20^ +28.8 (*c* 0.20, MeOH). IR (KBr) ν_max_ cm^−1^: 3429, 2952, 2848, 1657, 1453, 1386, 1119, 981, 882; ^1^H NMR (500 MHz, CDCl_3_) and ^13^C NMR (125 MHz, CDCl_3_) data, see Table 1; HRESIMS *m/z*: 477.3340 [M + Na]^+^ (calcd for C_30_H_46_O_3_Na, 477.3345).

### Bipolenin F (**3**)

White powder; [α]_D_^17^ +1.6 (*c* 0.20, MeOH). ^1^H NMR (600 MHz, CDCl_3_) and ^13^C NMR (150 MHz, CDCl_3_) data, see Table 1; HREIMS *m/z*: 280.2025 [M]^+^ (calcd for C_17_H_28_O_3_, 280.2038).

## Electronic supplementary material

Below is the link to the electronic supplementary material.

Supplementary material 1 (PDF 1499 kb)
