# Supplementary material for: Seco-sativene and Seco-longifolene Sesquiterpenoids from Cultures of Endophytic Fungus Bipolaris eleusines
Source: Nat Prod Bioprospect. 2017 Jan 6;7(1):147–50. doi: 10.1007/s13659-016-0116-4 (PMC5315672; doi:10.1007/s13659-016-0116-4)
Supplement: Supplementary file 1 — Supplementary material 1 (PDF 1499 kb) [file 13659_2016_116_MOESM1_ESM.pdf]

Supporting information for

Seco-sativene and seco-longifolene sesquiterpenoids from cultures of endophytic fungus *Bipolaris eleusines*

Man-Si Yang[a,b], Xiao-Yue Cai[b], Yuan-Yuan He[b], Meng-Ying Lu[b], Shuang Liu[b], Wen-Xiang Wang[a], Zheng-Hui Li[b], Hong-Lian Ai[b,*] and Tao Feng[b,*]

[a]*School of Agriculture and Biological Technic, Yunnan Agricultural University, Kunming 650201, China*

[b]*College of Pharmacy, South-Central University for Nationalities, Wuhan 430074, China*

Content

Figure 1S-7S, NMR spectra for compound **1**

Figure 8S-14S, MS and NMR spectra for compound **2**

Figure 15S-18S, MS and NMR spectra for compound **3**

[*]Corresponding author. College of Pharmacy, South-Central University for Nationalities, Wuhan 430074, China Tel.: +86 27 67841275; fax: +86 27 67841275.

E-mail addresses: 2015050@mail.scuec.edu.cn (H. L. Ai); tfeng@mail.scuec.edu.cn (T. Feng).

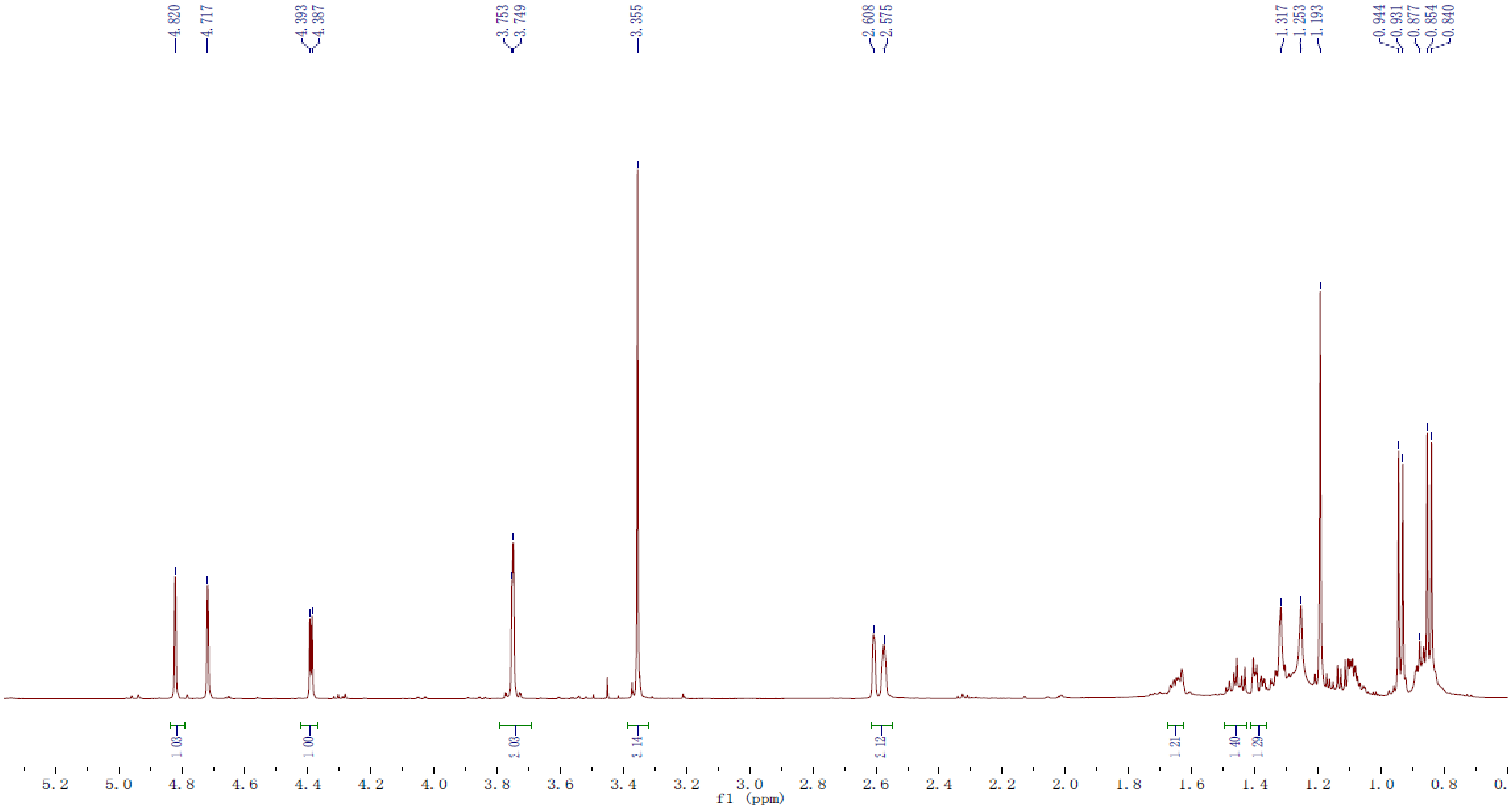

Figure 1S. ^{1}H NMR spectrum of compound **1**.

Figure 2S. ^{13}C NMR spectrum of compound **1**.

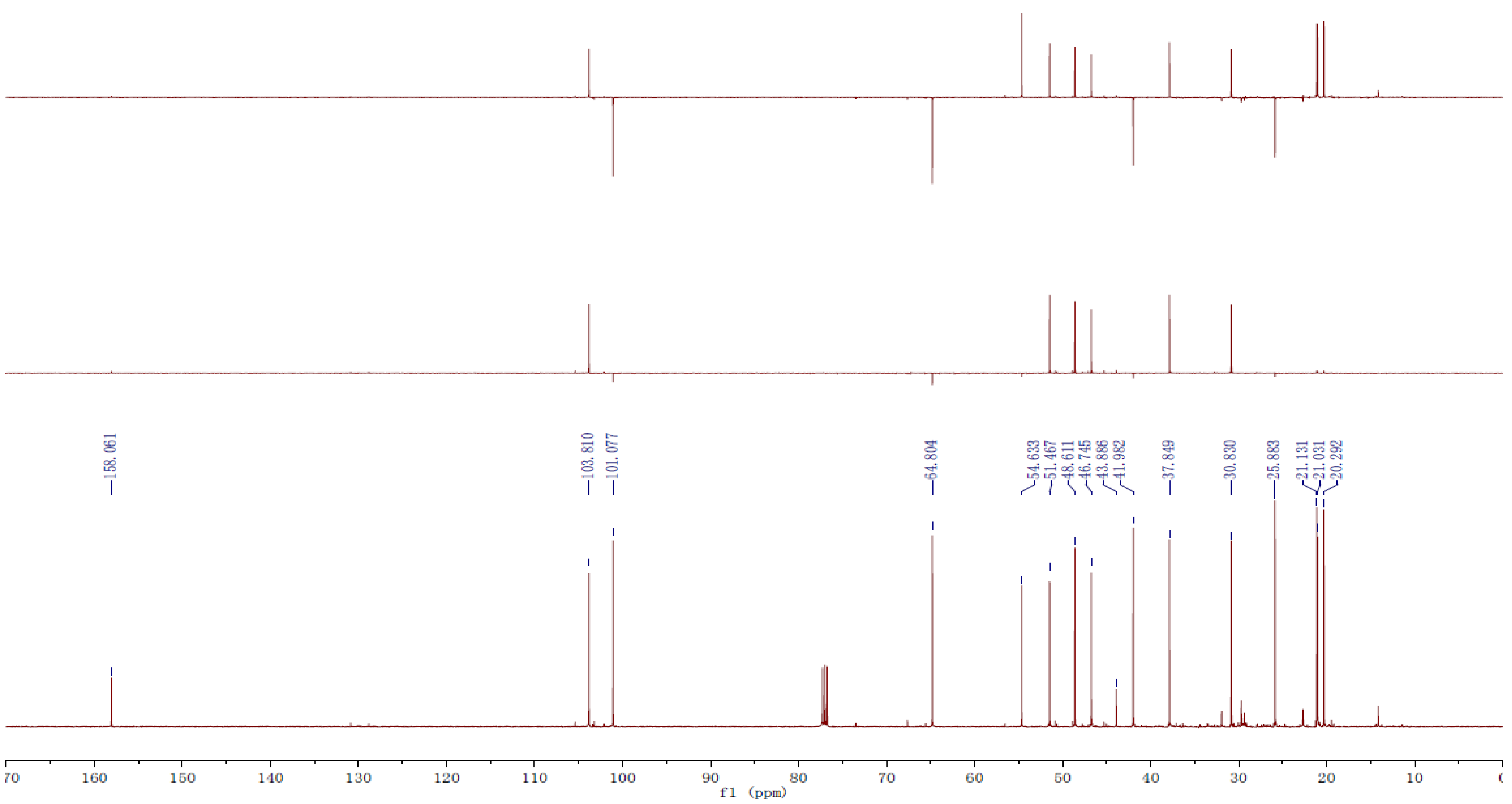

Figure 3S. HSQC spectrum of compound **1**.

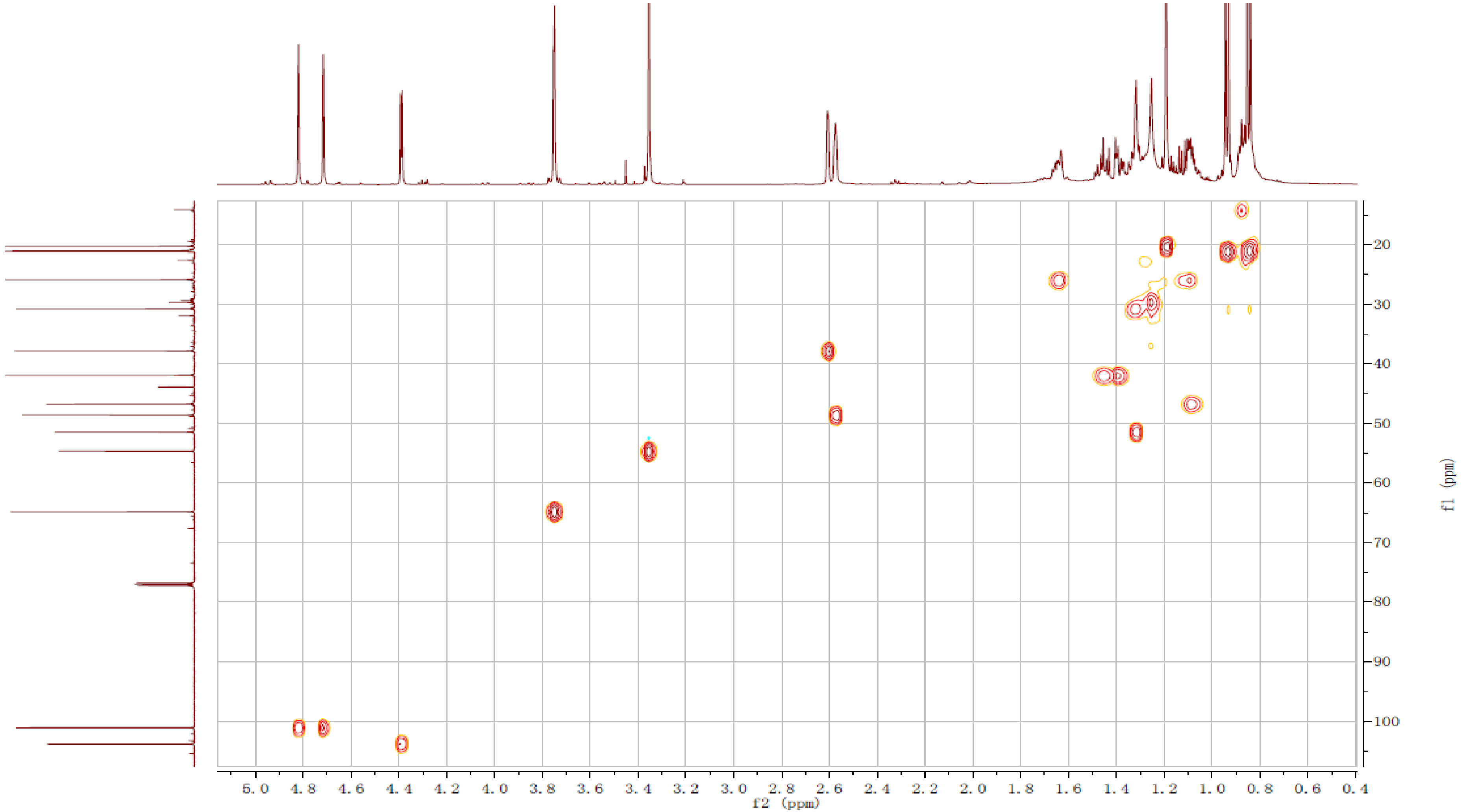

Figure 4S. HMBC spectrum of compound **1**.

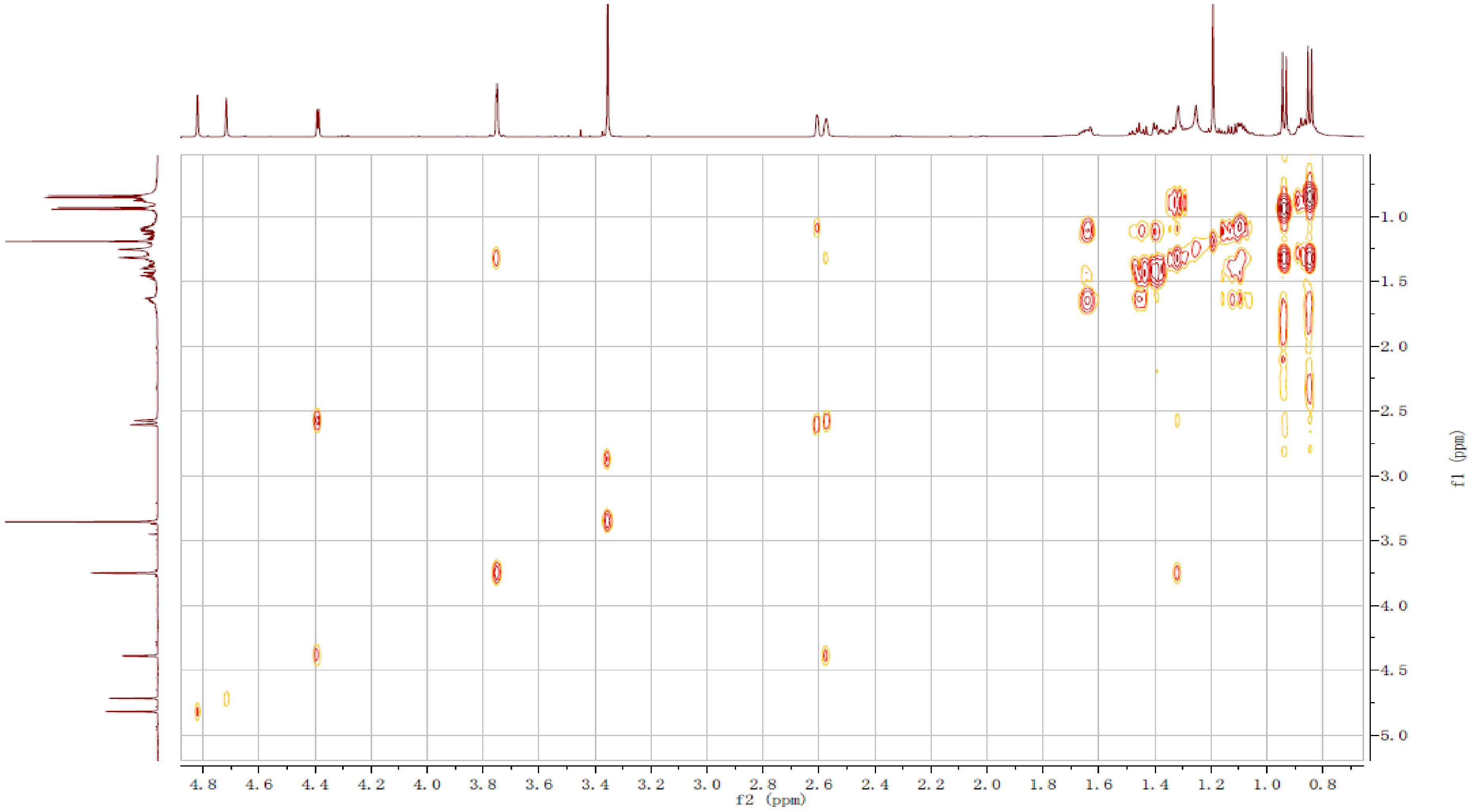

Figure 5S. ^{1}H-^{1}H COSY spectrum of compound **1**.

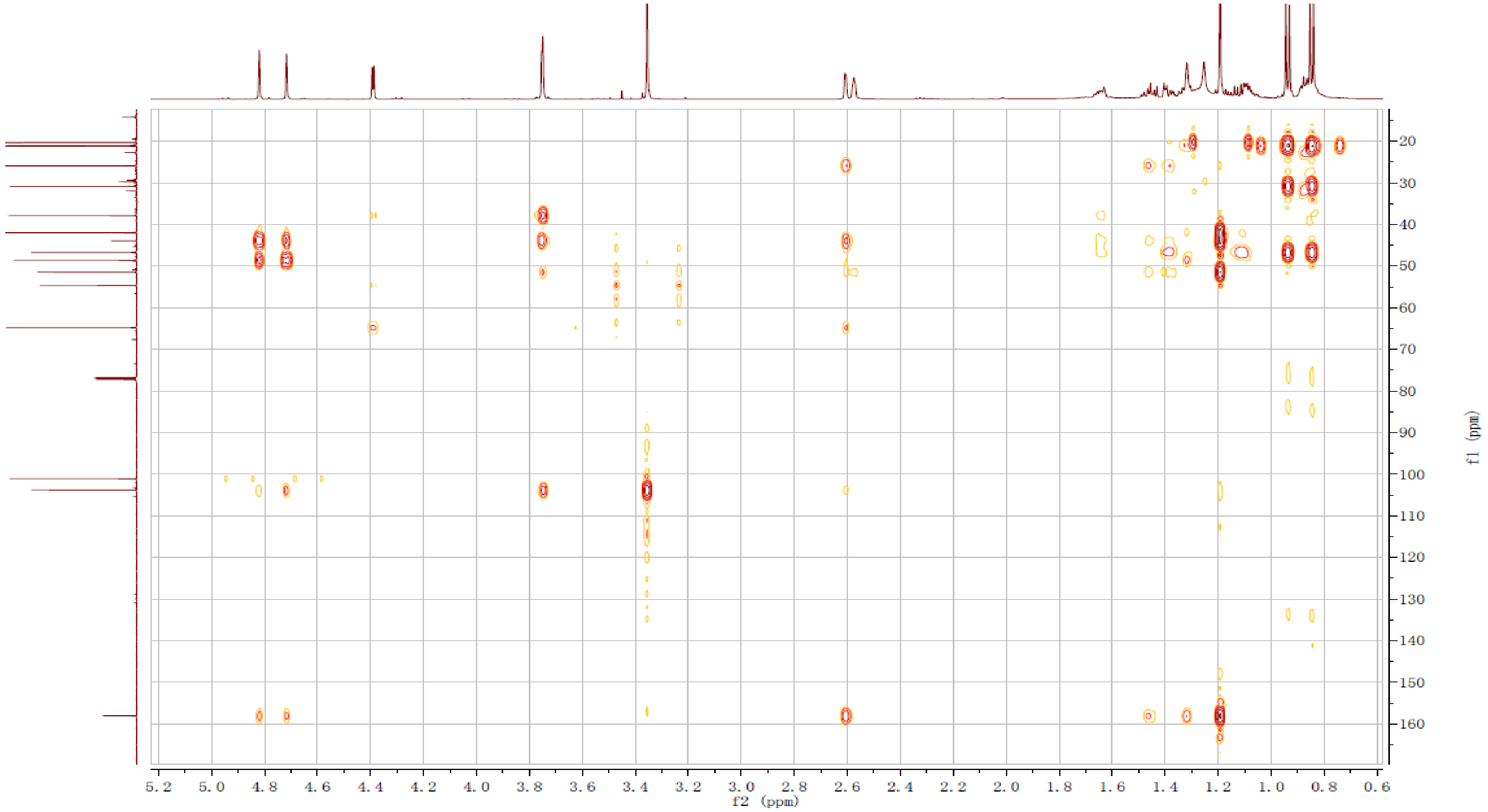

Figure 6S. ROESY spectrum of compound **1**.

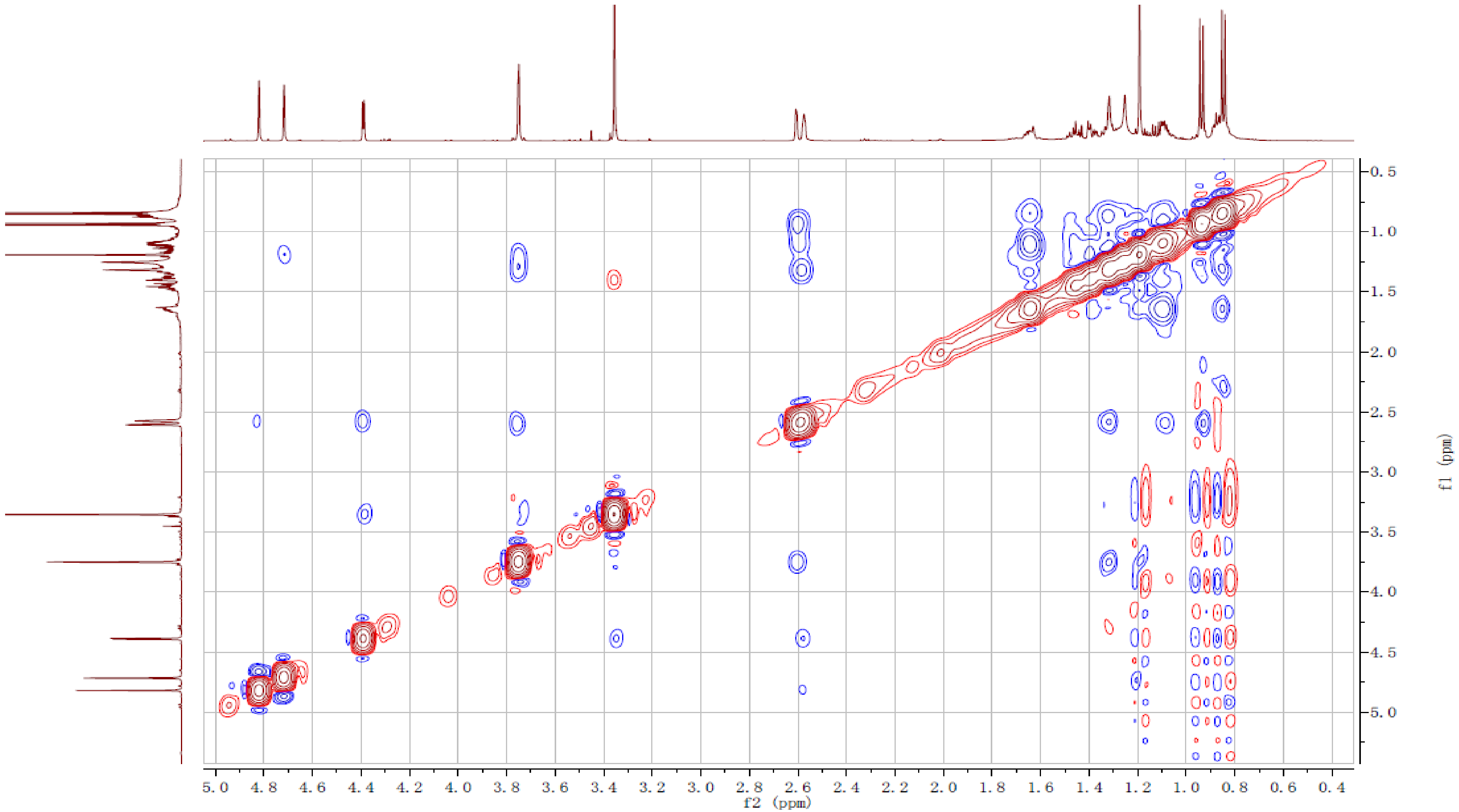

Figure 7S. ROESY spectrum of compound **1**.

User Spectra

Fragmentor Voltage	Collision Energy	Ionization Mode
175	0	ESI

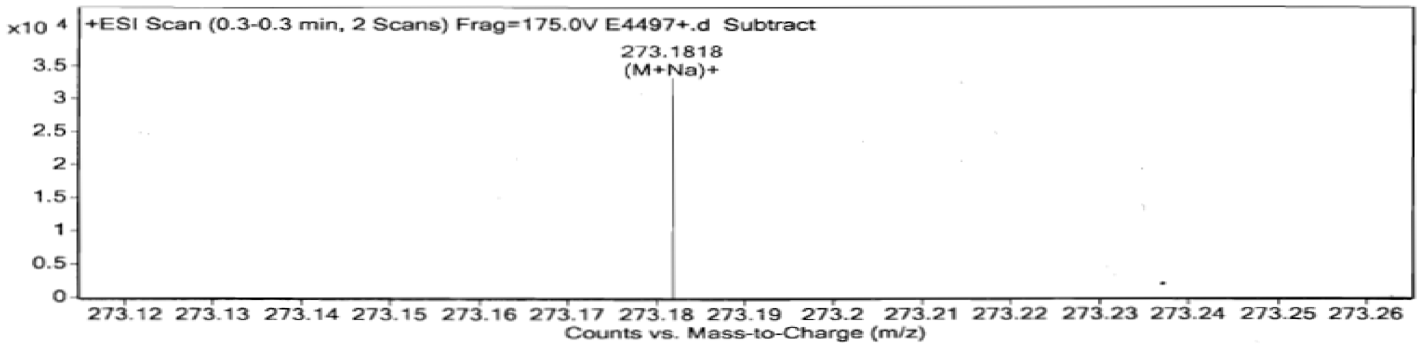

Peak List

m/z	z	Abund	Formula	Ion
145.0642	1	35442.09		
149.0228	1	284415.56		
193.0485	1	37937.07		
219.1736	1	47932.55		
230.0803	1	92945.53		
273.1818	1	33258.57	C16 H26 O2	(M+Na)+
301.1408	1	159117.48		
361.1617	1	73630.57		

Formula Calculator Element Limits

Element	Min	Max
C	3	60
H	0	120
O	0	30

Formula Calculator Results

Formula	CalculatedMass	CalculatedMz	Mz	Diff. (mDa)	Diff. (ppm)	DBE
C16 H26 O2	250.1933	273.1825	273.1818	0.7	2.7	4.0000

--- End Of Report ---

Figure 8S. ^{1}H NMR spectrum of compound **2**.

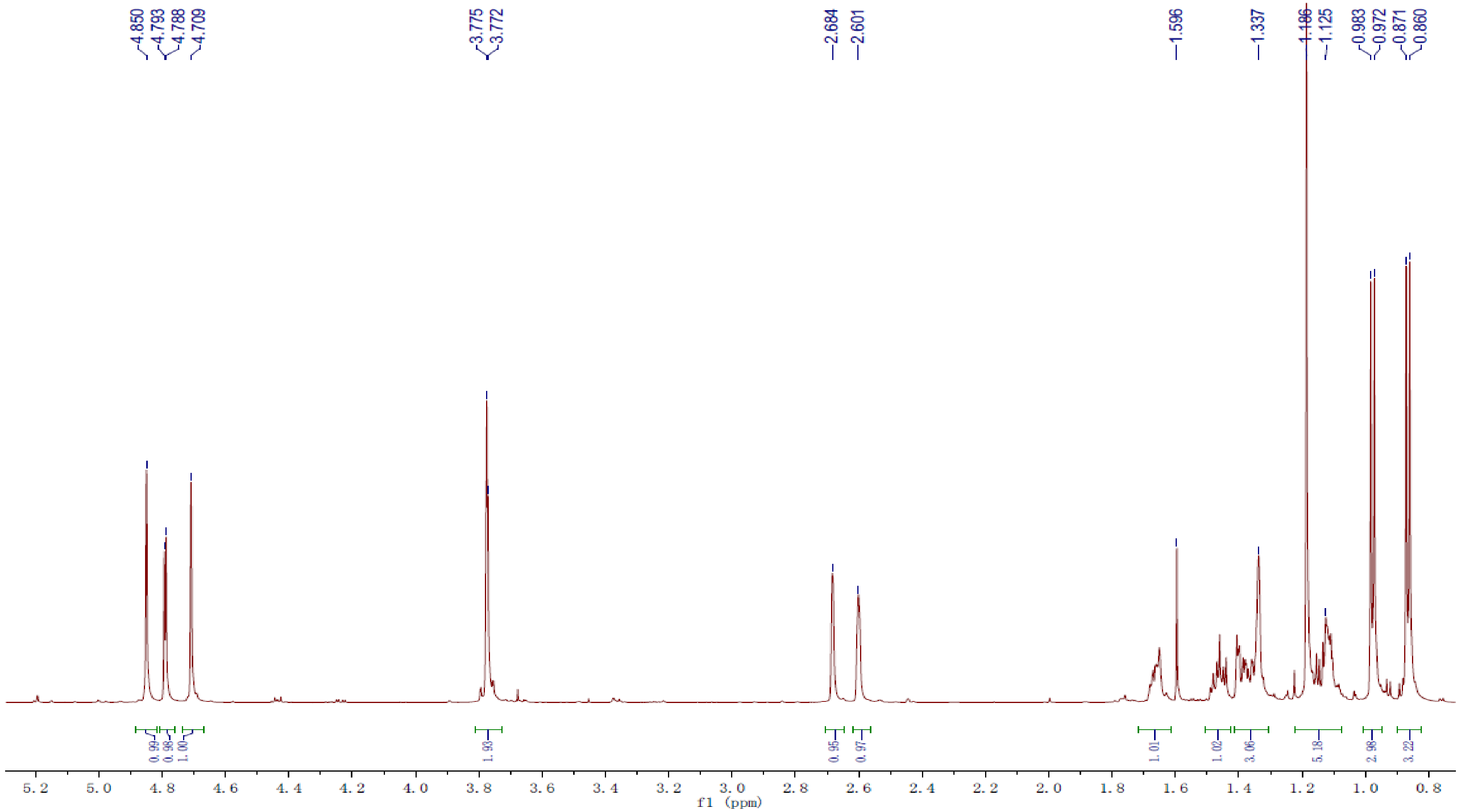

Figure 9S. ^{13}C NMR spectrum of compound **2**.

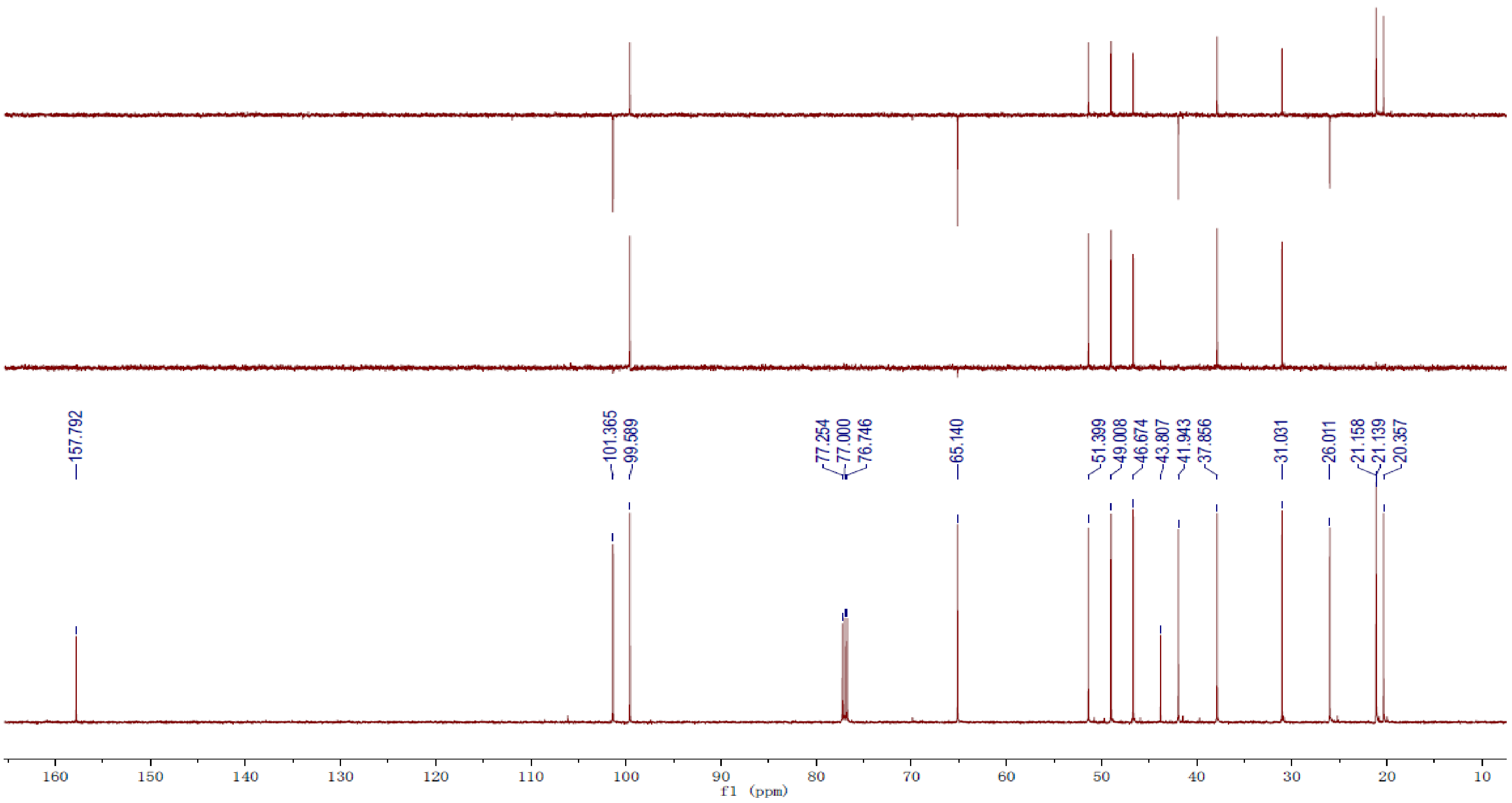

Figure 10S. HSQC spectrum of compound **2**.

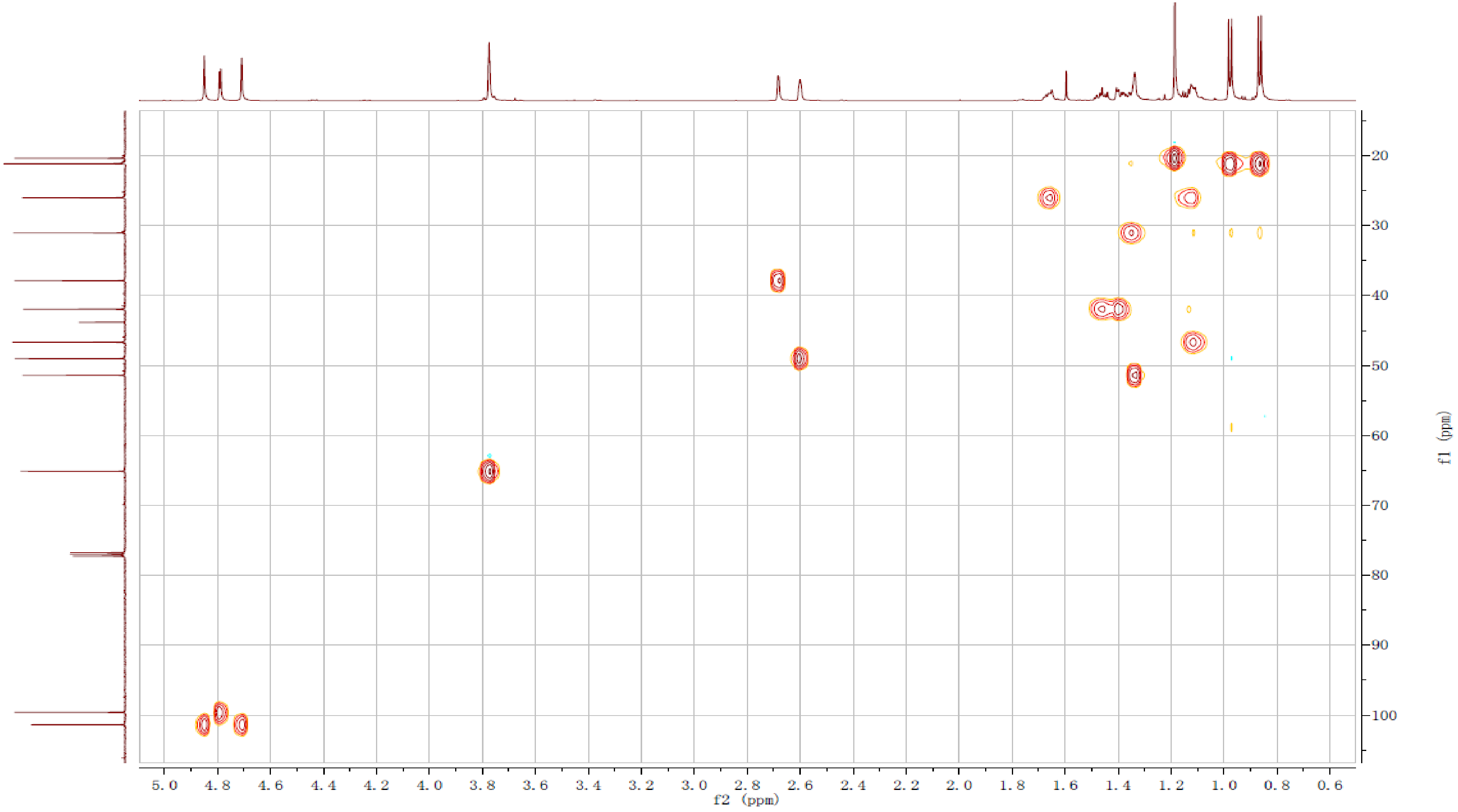

Figure 11S. HMBC spectrum of compound **2**.

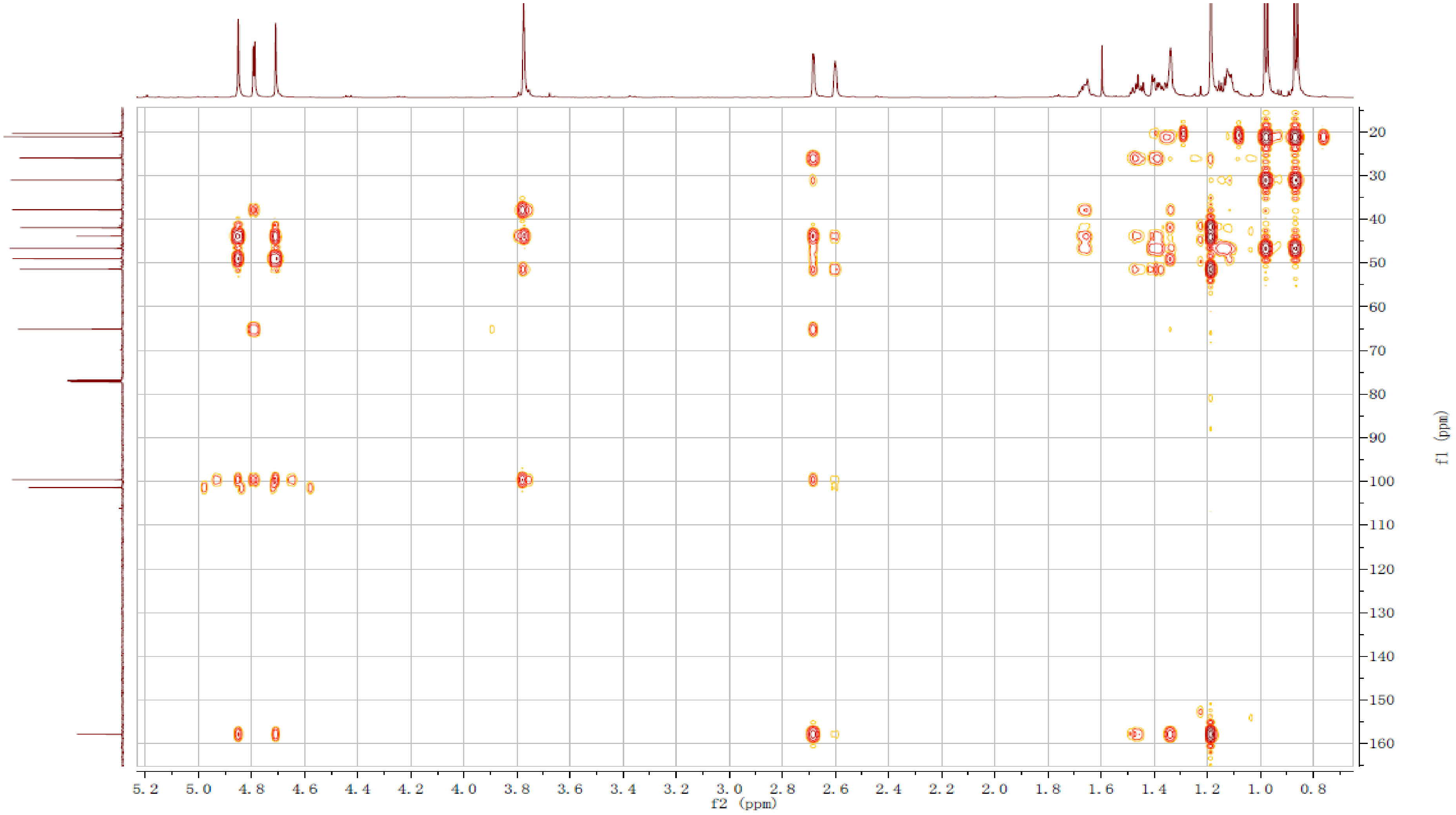

Figure 12S. ^{1}H-^{1}H COSY spectrum of compound **2**.

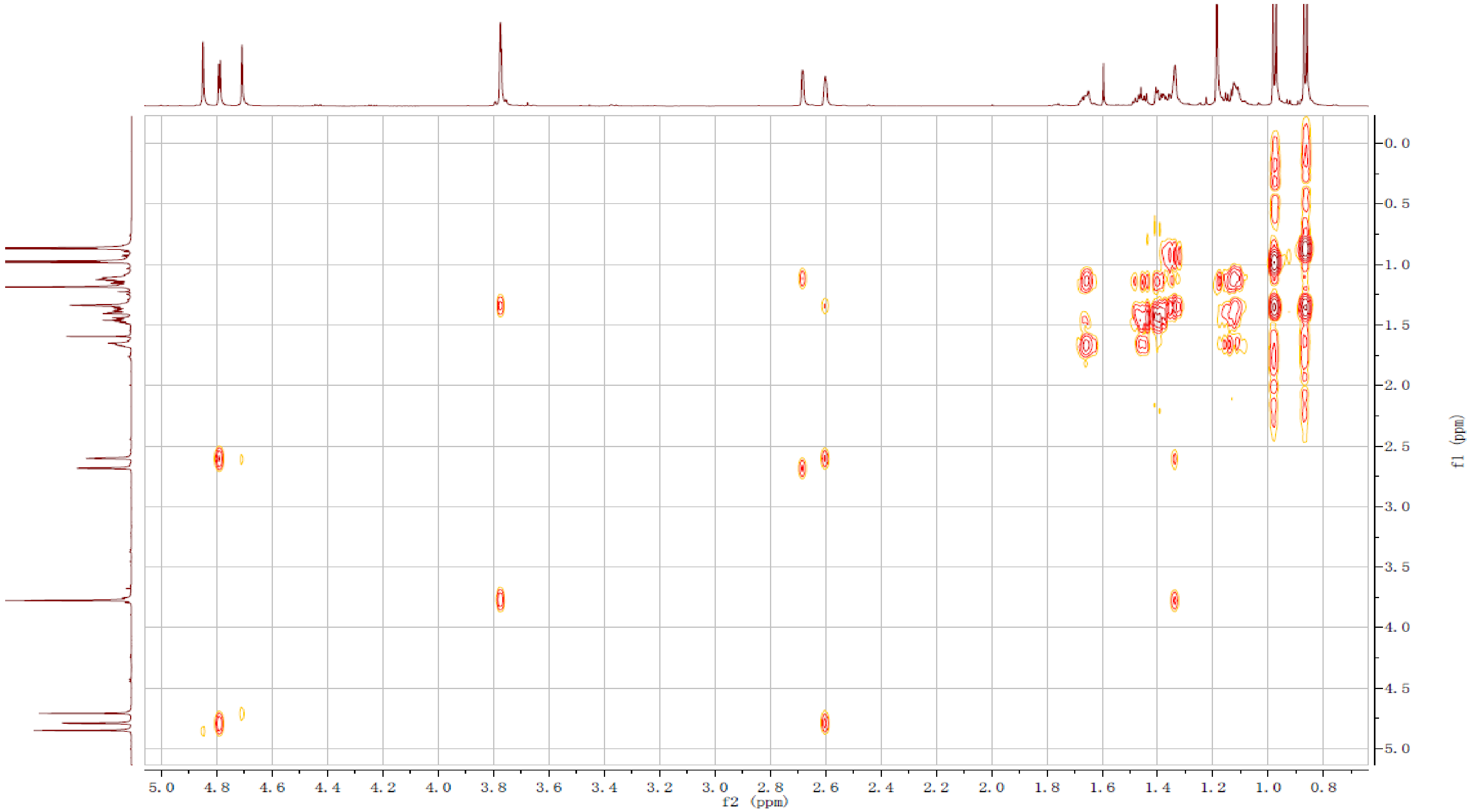

Figure 13S. ROESY spectrum of compound **2**.

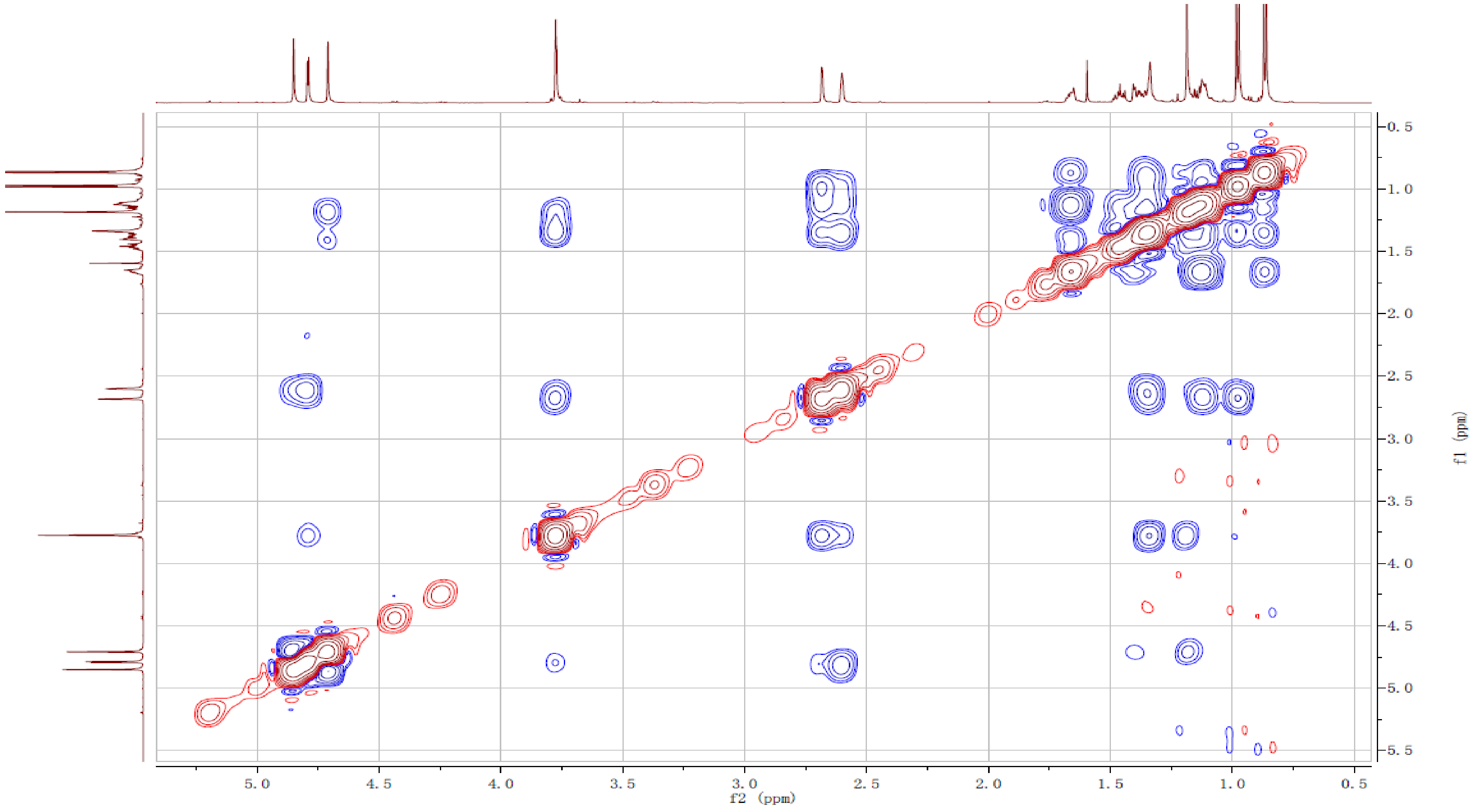

Figure 14S. HRESIMS of compound **2**.

User Spectra

Fragmentor Voltage	Collision Energy	Ionization Mode
200	0	ESI

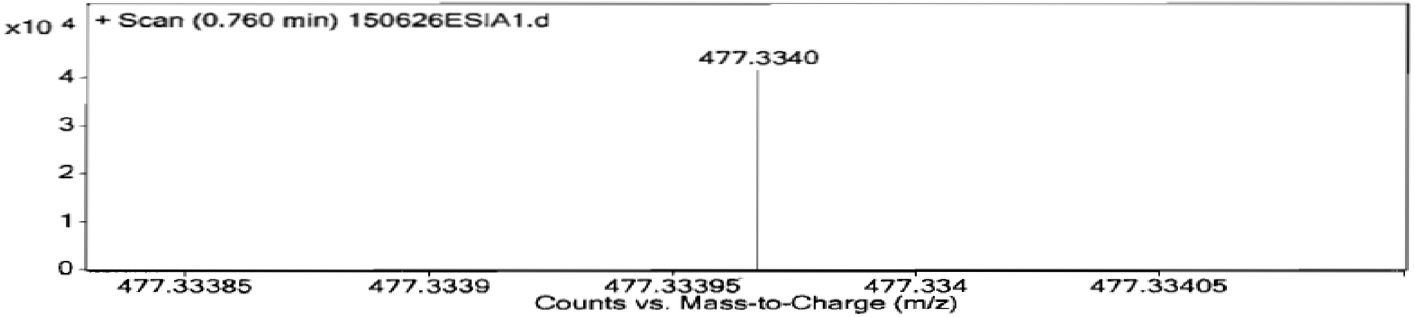

Peak List

m/z	z	Abund	Formula	Ion
121.0509	1	41585.08		
166.0836	1	28182.11		
219.1743	1	16320.12		
232.1123	1	326333.97		
233.1152	1	49875.4		
284.3311	1	57408.71		
312.3626	1	40590		
477.334	1	41517.39	C30 H46 Na O3	M+
507.3444	1	14160.4		
922.0098	1	57358.83		

Formula Calculator Element Limits

Element	Min	Max
C	0	200
H	0	400
O	0	9
Na	1	1

Formula Calculator Results

Formula	CalculatedMass	CalculatedMz	Mz	Diff. (mDa)	Diff. (ppm)	DBE
C30 H46 Na O3	477.3345	477.3339	477.3340	0.1	0.3	7.5000

--- End Of Report ---

Figure 15S. ^{1}H NMR spectrum of compound **3**.

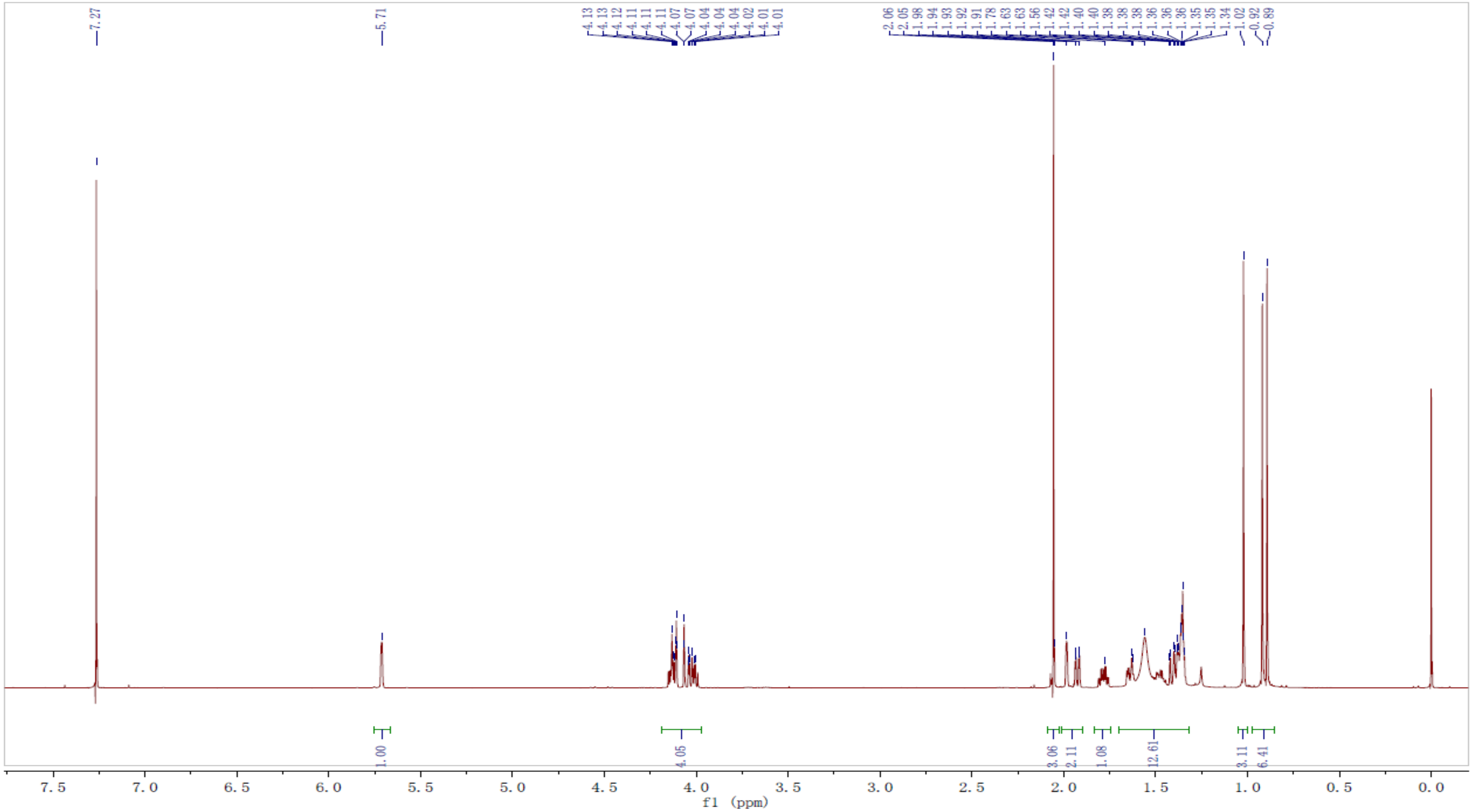

Figure 16S. ^{13}C NMR spectrum of compound **3**.

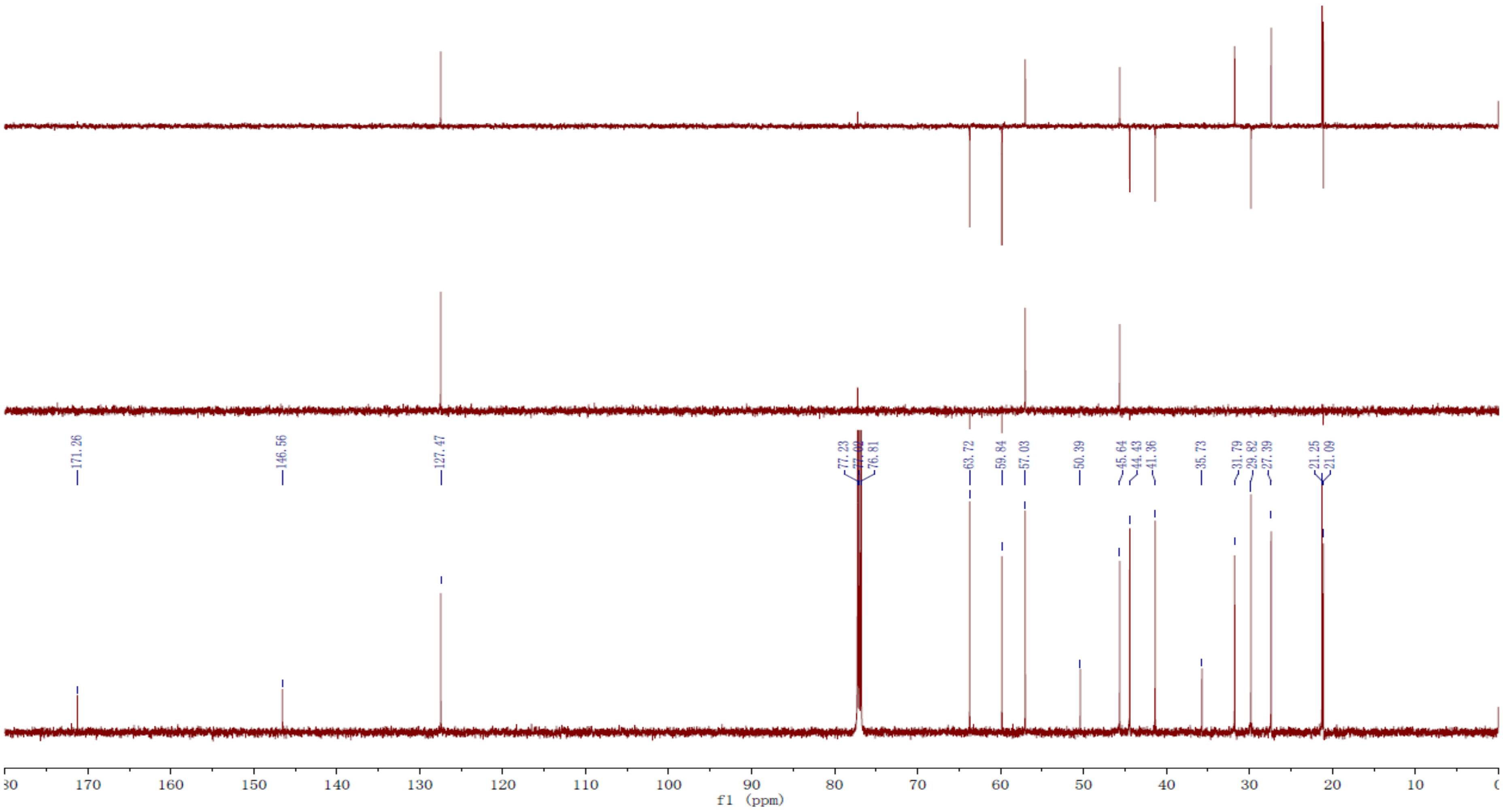

Figure 17S. HSQC spectrum of compound **3**.

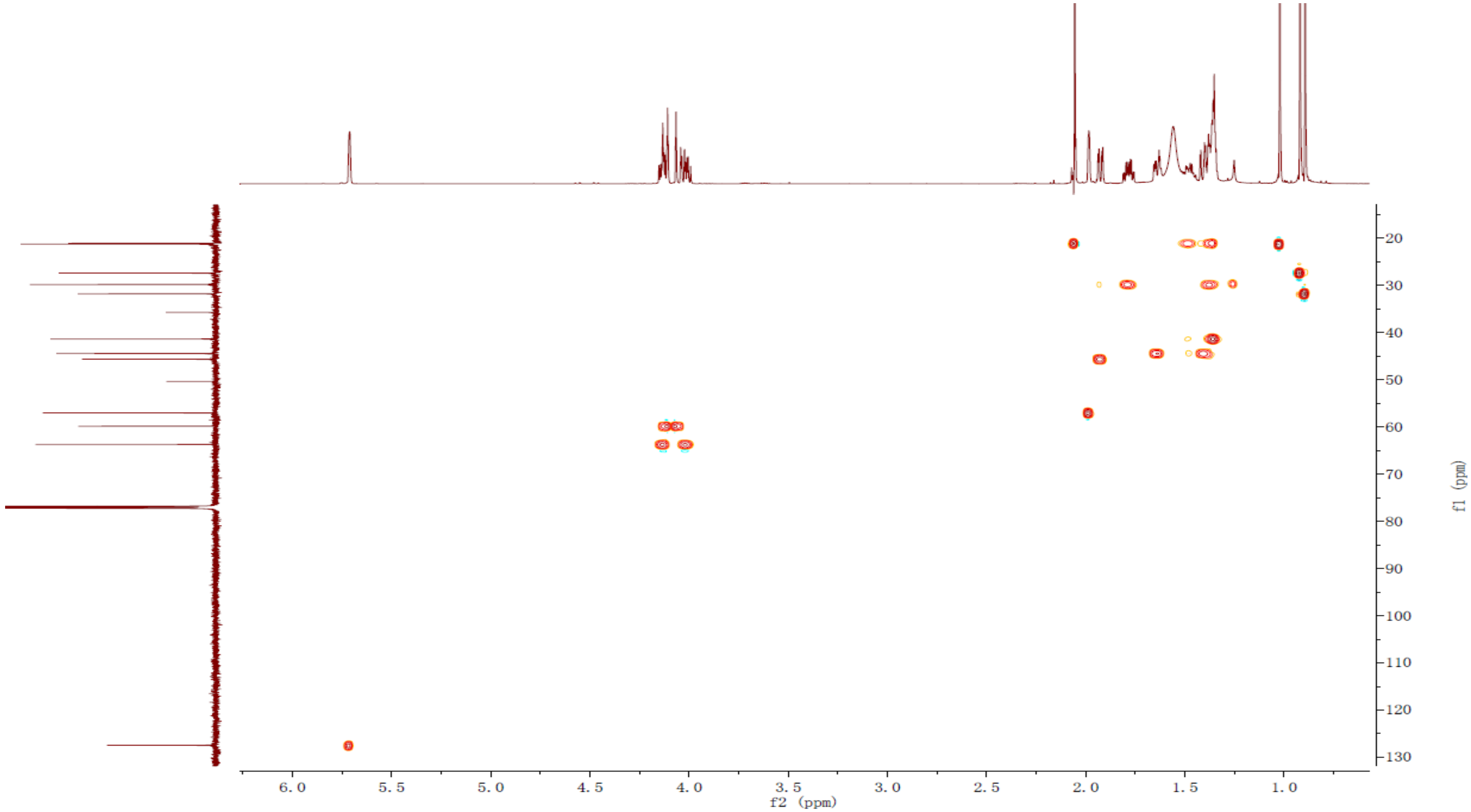

Figure 18S. HREIMS of compound **3**.

Elemental Composition Report

Single Mass Analysis

Tolerance = 10.0 PPM / DBE: min = -10.0, max = 120.0
Selected filters: None

Monoisotopic Mass, Odd and Even Electron Ions
15 formula(e) evaluated with 1 results within limits (up to 51 closest results for each mass)
Elements Used:
C: 0-200 H: 0-400 O: 2-4

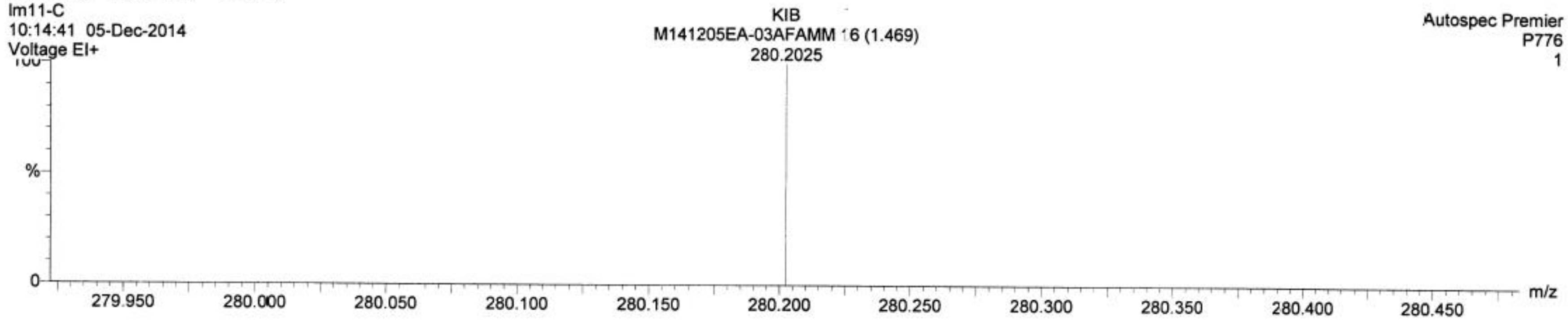

Minimum:				-10.0		
Maximum:		200.0	10.0	120.0		
Mass	Calc. Mass	mDa	PPM	DBE	i-FIT	Formula
280.2025	280.2038	-1.3	-4.6	4.0	5546026.0	C17 H28 O3
